# Supplementary material for: An Improved, Assay Platform Agnostic, Absolute Single Sample Breast Cancer Subtype Classifier
Source: Cancers (Basel). 2020 Nov 25;12(12):3506. doi: 10.3390/cancers12123506 (PMC7761033; doi:10.3390/cancers12123506)
Supplement: Supplementary file 1 [file cancers-12-03506-s001.pdf]

Supplementary Material: An Improved, Assay Platform Agnostic, Absolute Single Sample Breast Cancer Subtype Classifier

Mi-kyoung Seo, Soonmyung Paik and Sangwoo Kim

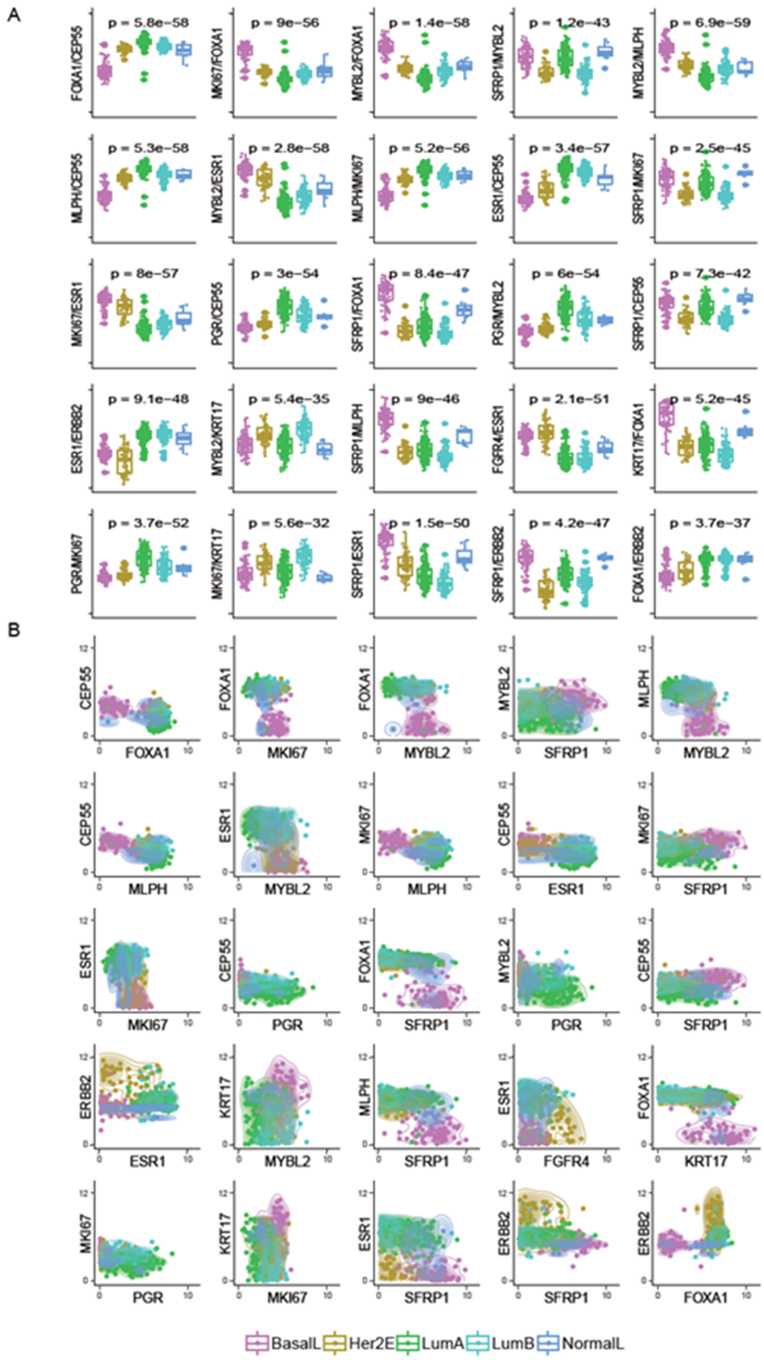

Figure S1. Biological relevance of the pairwise gene expression ratios (PGERs). (**A**) Boxplots of top 25 most important features PGERs. The Kruskal-Wallis test was performed to determine whether each feature was significantly different among the subtypes. The five colors represent the five subtypes. (**B**) Clustering in two-dimensional gene-pair space. Each subtype is closely enriched by the expression values of two genes, indicating that the use of two genes can be a feature that can classify subtypes. Five color dots represent five subtypes.

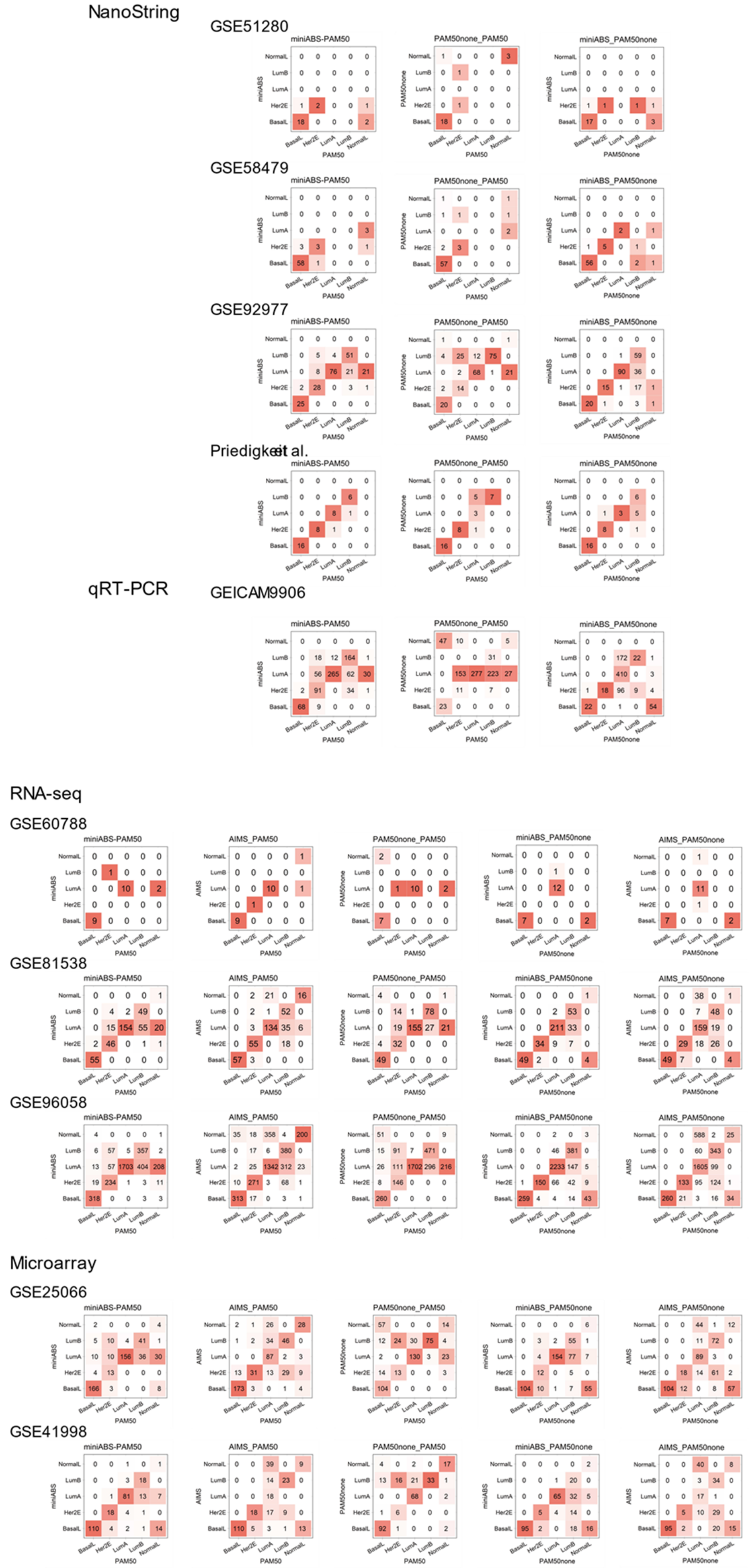

Figure S2. Confusion matrix for validation dataset. PAM50none is a subtype of the sample identified by the genefu package using the "none" option. * BasalL = Basal-like subtype; Her2E = HER2-enriched subtype; LumA = Luminal A subtype; LumB = Luminal B subtype; NormalL = Normal-like subtype.

Table S1. Dataset characteristics.

**Cohort**	Dataset	Technology	# Samples					
			Basal-like	Her2E	LumA	LumB	Normal-like	Total
**Discovery**	TCGA BRCA Nature 2012 [1]	RNA-seq	76	50	194	105	7	432
Validation	GSE60788 [2] (SCAN-B)	RNA-seq	9	1	10	0	2	22
	GSE81538 [3]	RNA-seq	57	65	156	105	22	405
	GSE96058 [3]	RNA-seq	360	348	1709	767	225	3409
	GSE25066 [4]	Affymetrix array	189	37	160	78	44	508
	GSE41998 [5]	Affymetrix array	110	23	91	33	22	279
	GSE51280 [6]	NanoString	19	2	0	0	3	24
	GSE58479 [7] (GEICAM2006-3)	NanoString	61	4	0	0	4	69
	GSE92977 [8]	NanoString	27	41	80	76	22	246
	Priedigkeit et al. [9]	NanoString	16	8	9	7	0	40
	GIECAM9906 [10]	qRT-PCR	70	174	277	261	32	814
Total			994	753	2686	1432	383	6248

* Her2E = HER2-enriched subtype; LumA = Luminal A subtype; LumB = Luminal B subtype

Table S2. Full list of ssDEGs.

**Basal-like ssDEGs**		**Her2E ssDEGs**		**LumA ssDEGs**		LumB ssDEGs		Normal-like ssDEGs	
Symbol	P value	Symbol	P value	Symbol	P value	Symbol	P value	Symbol	P value
MLPH	3.99 x 10-41	FGFR4	4.60x10-19	CEP55	2.37x10-50	KRT17	1.98x10-20	ERBB2	5.40x10-3
FOXA1	9.63x10-40	GRB7	5.60x10-16	MYBL2	7.03x10-49	SFRP1	7.65x10-20	KRT14	2.32x10-3
FOXC1	1.01x10-38	ERBB2	1.43x10-15	MELK	3.79x10-47	KRT14	5.66x10-18	KRT5	2.95x10-3
ESR1	1.04x10-35	BCL2	5.20x10-14	KIF2C	7.52x10-47	KRT5	1.58x10-17	MIA	3.19x10-3
NAT1	1.38x10-35	ESR1	3.63x10-12	ANLN	1.20x10-46	EGFR	1.12x10-16	SFRP1	4.85x10-3
CCNE1	9.46x10-34	SLC39A6	3.19x10-11	CDC20	1.78x10-45	MIA	6.18x10-16		
CXXC5	3.31x10-33	RRM2	3.34x10-11	BIRC5	5.34x10-45	FOXC1	6.51x10-16		
SLC39A6	1.95x10-31	TMEM45B	9.78x10-11	UBE2C	8.93x10-45	CDC6	1.30x10-12		
CDC20	3.37x10-31	PGR	8.18x10-10	NDC80	9.23x10-45	CCNB1	3.96x10-12		
GPR160	9.86x10-31	MAPT	4.07x10-9	UBE2T	1.70x10-44	CDH3	6.06x10-12		
MAPT	2.46x10-30	CDC6	4.25x10-9	EXO1	1.86x10-44	ESR1	7.74x10-12		
ORC6L	7.28x10-29	MIA	2.41x10-7	NUF2	6.13x10-44	UBE2T	1.15x10-11		
SFRP1	1.16x10-28	MYBL2	3.32x10-7	MKI67	1.86x10-43	CXXC5	1.23x10-11		
PGR	4.69x10-28	UBE2T	3.26x10-6	PTTG1	2.16x10-42	BIRC5	4.97x10-11		
TMEM45B	7.01x10-28	CEP55	3.99x10-6	CDC6	1.84x10-41	MKI67	9.52x10-10		
PHGDH	3.58x10-27	MKI67	4.26x10-6	RRM2	4.05x10-41	BLVRA	5.92x10-09		
NDC80	6.27x10-27	UBE2C	8.87x10-6	CENPF	4.33x10-40	RRM2	6.84x10-9		
MIA	1.64x10-26	ANLN	1.01x10-5	CCNE1	3.41x10-39	CENPF	6.88x10-9		
KIF2C	5.34x10-26	EXO1	1.13x10-5	ORC6L	4.27x10-37	SLC39A6	7.45x10-9		
MELK	4.85x10-25	MDM2	1.27x10-5	CCNB1	1.10x10-35	MYBL2	1.26x10-8		
CDH3	6.45x10-25	MYC	1.92x10-5	TYMS	9.13x10-30	NUF2	1.55x10-8		
CEP55	1.38x10-23	GPR160	2.08x10-5	PGR	4.93x10-28	UBE2C	3.44x10-8		
EGFR	1.99x10-23	NAT1	2.52x10-5	MAPT	5.19x10-24	TYMS	6.55x10-8		
NUF2	2.14x10-23	MELK	2.56x10-5	BCL2	3.25x10-23	CEP55	7.10x10-8		
ANLN	3.31x10-23	BAG1	2.66x10-5	NAT1	1.11x10-22	EXO1	2.29x10-7		
BLVRA	1.25x10-22	SFRP1	3.57x10-5	MLPH	1.86x10-19	FOXA1	2.44x10-7		
EXO1	2.04x10-20	FOXC1	4.54x10-5	PHGDH	4.29x10-19	NDC80	2.68x10-7		
CENPF	5.56x10-20	CCNE1	1.00x10-4	SLC39A6	3.39x10-18	PTTG1	5.61x10-7		
ERBB2	1.30x10-19	CCNB1	1.19x10-4	ESR1	3.10x10-17	KIF2C	5.85x10-7		
ACTR3B	4.07x10-19	PTTG1	1.23x10-4	FOXA1	1.11x10-12	ANLN	7.01x10-7		
BCL2	4.75x10-19	CDC20	1.89x10-4	BAG1	4.13x10-12	MDM2	2.18x10-6		
PTTG1	7.29x10-19	CXXC5	3.40x10-4	CXXC5	5.82x10-10	MELK	2.53x10-5		
BIRC5	1.11x10-18	PHGDH	8.03x10-4	TMEM45B	8.93x10-7	MLPH	1.09x10-4		
TYMS	1.65x10-18	KRT14	8.65x10-4	GPR160	1.71x10-6	CDC20	8.90x10-4		
MYBL2	9.25x10-18	ORC6L	9.71x10-4	KRT14	1.97x10-6	NAT1	2.55x10-3		
KRT17	1.06x10-17	KIF2C	1.46x10-3	FGFR4	5.92x10-5	ACTR3B	3.39x10-3		
UBE2C	1.72x10-16			GRB7	1.62x10-4	MAPT	4.64x10-3		
MKI67	5.29x10-15			MDM2	4.41x10-4				

UBE2T	3.51x10-12	CDH3	1.81x10-3
MYC	3.54x10-12		
KRT5	8.36x10-11		
RRM2	3.91x10-9		
MDM2	9.81x10-9		
CCNB1	1.24x10-8		
MMP11	2.05x10-8		
BAG1	6.23x10-08		
KRT14	1.12x10-7		
CDC6	3.61x10-7		

P values were determined using Wilcoxon rank-sum test. *P* values less than 0.005 were regarded as statistically significant. The colors denote over- (red) or down- (blue) regulated genes compared to median expression of the gene between the given subtype and the remaining subtypes.

Table S3. Statistical significance of seed genes.

	**ESR1**		**ERBB2**		PGR		MKI67	
ssDEGs	P value	FDR	P value	FDR	P value	FDR	P value	FDR
Basal-like	1.04x10-35	6.47x10-33	1.30x10-19	1.96x10-18	4.69x10-28	2.93x10-26	5.29x10-15	4.47x10-14
Her2E	3.63x10-12	9.53x10-10	1.43x10-15	1.62x10-12	8.18x10-10	7.34x10-8	4.26x10-6	6.75x10-5
LumA	3.10x10-17	8.53x10-16	5.23x10-2	8.45x10-2	4.93x10-28	5.03x10-26	1.86x10-43	7.95x10-41
LumB	7.74x10-12	5.18x10-10	3.91x10-1	5.17x10-1	8.28x10-2	1.54x10-1	9.52x10-10	2.98x10-8
Normal-like	2.01x10-1	5.68x10-1	5.38x10-4	2.42x10-1	6.47x10-1	8.61x10-1	1.18x10-1	4.71x10-1

P values were determined using Wilcoxon rank-sum test. * Her2E = HER2-enriched subtype; LumA = Luminal A subtype; LumB = Luminal B subtype; ssDEG = subtype-specific Differentially Expressed Gene.

Table S4. Comparison of four machine learning algorithms.

**ML**	**α value**						
	**0.00**	**0.01**	**0.05**	**0.10**	**0.15**	**0.20**	**1.00**
			1st-level				
CART	76.90	76.95	76.96	77.03	77.03	77.06	77.54
RF	83.15	83.23	83.20	83.26	83.24	83.15	**83.45**
SVM	77.24	77.27	77.26	77.33	77.42	77.41	76.37
NB	80.81	80.81	80.89	80.94	81.02	81.05	80.55
			2nd-level				
CART	82.55	82.55	82.55	82.59	82.58	82.58	81.21
RF	87.82	87.84	87.88	87.86	87.91	87.85	**88.04**
SVM	75.85	75.81	75.72	75.65	75.51	75.35	73.00
NB	85.06	85.06	85.16	84.96	84.94	84.95	82.84
			3rd-level				
CART	79.41	79.41	79.40	79.42	79.45	79.44	78.30
RF	86.87	86.86	86.93	86.87	86.86	86.95	**87.03**
SVM	74.57	74.56	74.57	74.49	74.44	74.37	73.01
NB	83.69	83.76	83.92	83.81	83.94	83.85	83.28
			4th-level				
CART	80.57	80.57	80.57	80.57	80.57	80.59	80.88
RF	87.40	87.36	87.37	87.46	**87.50**	87.42	87.12
SVM	73.76	73.74	73.71	73.68	73.63	73.62	72.35
NB	85.43	85.48	85.51	85.52	85.65	85.56	84.32
			5th-level				
CART	80.55	80.55	80.55	80.54	80.54	80.55	80.89
RF	87.22	87.30	87.34	87.30	**87.40**	87.33	87.20
SVM	73.78	73.74	73.71	73.65	73.59	73.52	72.10
NB	85.49	85.54	85.56	85.53	85.80	85.72	84.21

For each input gene set, the RF models showed the best performance, regardless of the α value, among the four algorithms. The model with the 2nd-level gene set achieved the highest accuracy (88.04%) among all models. The number in bold corresponds to the highest accuracy in each input gene set.

* ML=Machine Learning; SVM=Support Vector Machine; CART=Classification and Regression Tree; RF=Random Forest; NB=Naïve Bayes

Table S5. Analysis of model accuracy depending on different gene set refinement strategies.

# Total genes	# Level of ssDEGs	ML algorithm	Best Accuracy	Gene set
9	1st	RF	85.23	ESR1 PGR ERBB2 MKI67 MLPH NUDT6 CEP55 TRIM29 AK5
14	2nd	RF	87.35	ESR1 PGR ERBB2 MKI67 MLPH NUDT6 CDC45 TRIM29 AK5 FOXA1 FGFR4 MYBL2 TIMELESS CAPN6
19	3rd	RF	86.63	ESR1 PGR ERBB2 MKI67 MLPH NUDT6 CDC45 TRIM29 AK5 FOXA1 FGFR4 MYBL2 TIMELESS CAPN6 XBP1 TCAP CENPA ID4 CCDC88B
24	4th	RF	**87.84**	ESR1 PGR ERBB2 MKI67 MLPH NUDT6 CDC45 TRIM29 AK5 FOXA1 FGFR4 MYBL2 TIMELESS CAPN6 XBP1 TCAP CENPA ID4 CCDC88B AR FA2H CDCA8 KRT17 CCL14
29	5th	RF	87.14	ESR1 PGR ERBB2 MKI67 MLPH NUDT6 CDC45 TRIM29 AK5 FOXA1 FGFR4 MYBL2 TIMELESS CAPN6 XBP1 TCAP CENPA ID4 CCDC88B AR FA2H CDCA8 KRT17 CCL14 FOXC1 DBNDD2 CDCA5 TCF7L1 COL17A1

The green genes correspond to seed genes. The bold text corresponds to the highest accuracy among all models. These models were constructed using an "intrinsic gene set" instead of the PAM50 gene set, and the accuracy was slightly lower, even with the use of more genes than included in MiniABS. * RF = Random Forest; ML = Machine Learning; ssDEG = subtype-specific Differentially Expressed Gene.

Table S6. Analysis of model accuracy with ssDEGs from all genes.

# Total genes	# Level of ssDEGs	ML algorithm	Best Accuracy	Gene set
9	1st	RF	86.21	ESR1 PGR ERBB2 MKI67 MLPH NUDT6 CDC45 TRIM29 ABCF1
14	2nd	RF	88.01	ESR1 PGR ERBB2 MKI67 MLPH NUDT6 CDC45 TRIM29 ABCF1 FOXA1 FGFR4 MCM10 KCNMB1 ADAR
19	3rd	RF	**88.81**	ESR1 PGR ERBB2 MKI67 MLPH NUDT6 CDC45 TRIM29 ABCF1 FOXA1 FGFR4 MCM10 KCNMB1 ADAR XBP1 PMAIP1 CEP55 TIMELESS ADH1C
24	4th	RF	88.09	ESR1 PGR ERBB2 MKI67 MLPH NUDT6 CDC45 TRIM29 ABCF1 FOXA1 FGFR4 MCM10 KCNMB1 ADAR XBP1 PMAIP1 CEP55 TIMELESS ADH1C AR TCAP FOXM1 ID4 ADIG
29	5th	RF	88.16	ESR1 PGR ERBB2 MKI67 MLPH NUDT6 CDC45 TRIM29 ABCF1 FOXA1 FGFR4 MCM10 KCNMB1 ADAR XBP1 PMAIP1 CEP55 TIMELESS ADH1C AR TCAP FOXM1 ID4 ADIG TBC1D9 FA2H AURKB BBOX1 ADNP2

The green genes correspond to seed genes. The bold text corresponds to the highest accuracy among all models. These models were constructed using ssDEGs for all genes of RNA-seq libraries, instead of the PAM50 gene set, and the accuracy was slightly higher than that for the MiniABS even though more genes had to be used. * RF = Random Forest; ML = Machine Learning; ssDEG = subtype-specific Differentially Expressed Gene.

Table S7. Analysis of model accuracy without seed genes.

# Total genes	# Level of ssDEGs	ML algorithm	Best Accuracy	Gene set
5	1st	RF	79.67	MLPH FGFR4 CEP55 KRT17 ERBB2
10	2nd	RF	85.01	MLPH FGFR4 CEP55 KRT17 ERBB2 FOXA1 GRB7 MYBL2 SFRP1 KRT14
13	3rd	RF	84.56	MLPH FGFR4 CEP55 KRT17 ERBB2 FOXA1 GRB7 MYBL2 SFRP1 KRT14 FOXC1 MELK KRT5
17	4th	RF	87.32	MLPH FGFR4 CEP55 KRT17 ERBB2 FOXA1 GRB7 MYBL2 SFRP1 KRT14 FOXC1 MELK KRT5 ESR1 BCL2 KIF2C MIA
20	5th	RF	**87.39**	MLPH FGFR4 CEP55 KRT17 ERBB2 FOXA1 GRB7 MYBL2 SFRP1 KRT14 FOXC1 MELK KRT5 ESR1 BCL2 KIF2C MIA NAT1 ANLN EGFR

To investigate the effect of the addition of seed genes on the performance of the model, the models were constructed using ssDEGs derived from PAM50 without additional seed genes. Although the 5th gene set (20 genes) was used, the accuracy was lower than that of MiniABS. * RF = Random Forest; ML = Machine Learning; ssDEG = subtype-specific Differentially Expressed Gene.

Table S8. Performance in the validation dataset.

miniABS vs. PAM50										
**Performance**	**GSE60788**	**GSE81538**	**GSE96058**	**GSE25066**	**GSE41998**	**GSE51280**	**GSE58479**	**GSE92977**	**Priedigkeit**	**geicam9906**
Basal-like_Sensitivity	100	96.49	88.33	88.77	100	94.74	95.08	92.59	100	97.14
Her2E_Sensitivity	0	70.77	67.24	36.11	78.26	100	75	68.29	100	52.3
LumA_Sensitivity	100	98.72	99.65	97.5	89.01	NA	NA	95	88.89	95.67
LumB_Sensitivity	NA	46.67	46.54	53.25	54.55	NA	NA	68	85.71	63.08
Normal-like_Sensitivity	0	4.55	0.44	9.3	4.55	0	0	0	NA	0
Basal-like_Specificity	100	100	99.8	96.52	87.57	60	87.5	100	100	98.79
Her2E_Specificity	100	98.82	98.89	99.14	98.05	90.91	93.85	97.06	96.88	94.21
LumA_Specificity	83.33	63.86	59.88	74.93	88.83	100	95.65	69.7	96.77	72.39
LumB_Specificity	95.45	98	97.35	95.31	98.78	100	100	94.71	100	94.39
Normal-like_Specificity	100	100	99.87	99.57	99.61	100	100	100	100	100
Basal-like_Pos Pred Value	100	100	98.15	93.79	83.97	90	98.31	100	100	88.31
Her2E_Pos Pred Value	NA	92	87.31	76.47	78.26	50	42.86	82.35	88.89	71.09
LumA_Pos Pred Value	83.33	63.11	71.4	64.46	79.41	NA	NA	60.32	88.89	64.16
LumB_Pos Pred Value	NA	89.09	83.61	67.21	85.71	NA	NA	85	100	84.1
Normal-like_Pos Pred Value	NA	100	20	66.67	50	NA	NA	NA	NA	NA
Basal-like_Neg Pred Value	100	99.43	98.64	93.56	100	75	70	99.09	100	99.73
Her2E_Neg Pred Value	95.45	94.65	96.37	95.27	98.05	100	98.39	93.84	100	87.88
LumA_Neg Pred Value	100	98.76	99.41	98.47	94.35	NA	NA	96.64	96.77	97
LumB_Neg Pred Value	NA	84	86.25	91.86	94.19	NA	NA	87.03	97.06	84.47
Normal-like_Neg Pred Value	90.91	94.8	93.42	92.15	92.42	87.5	94.2	91.02	NA	96.06
Basal-like_Balanced Accuracy	100	98.25	94.07	92.64	93.79	77.37	91.29	96.3	100	97.97
Her2E_Balanced Accuracy	50	84.8	83.07	67.63	88.15	95.45	84.42	82.68	98.44	73.25
LumA_Balanced Accuracy	91.67	81.29	79.77	86.21	88.92	NA	NA	82.35	92.83	84.03

LumB_Balanced Accuracy	NA	72.33	71.95	74.28	76.66	NA	NA	81.35	92.86	78.74
Normal-like_Balanced Accuracy	50	52.27	50.16	54.43	52.08	50	50	50	NA	50

miniABS vs. PAM50none

**Performance**	**GSE60788**	**GSE81538**	**GSE96058**	**GSE25066**	**GSE41998**	**GSE51280**	**GSE58479**	**GSE92977**	**Priedigkeit**	**geicam9906**
Basal-like_Sensitivity	100	100	99.62	100	100	94.44	98.25	100	100	95.65
Her2E_Sensitivity	NA	94.44	97.4	41.38	71.43	100	100	93.75	88.89	100
LumA_Sensitivity	92.31	95.05	94.98	98.09	92.86	NA	100	97.83	100	60.38
LumB_Sensitivity	NA	56.99	65.24	38.19	23.81	0	0	51.3	50	70.97
Normal-like_Sensitivity	0	20	5	8.7	8.7	0	0	0	NA	0
Basal-like_Specificity	86.67	98.31	97.94	81.7	80.43	50	75	97.78	100	93.04
Her2E_Specificity	100	95.66	96.37	98.95	93.38	86.96	96.88	91.7	96.77	86.16
LumA_Specificity	100	81.97	85.63	74.57	82.3	100	98.51	76.47	83.78	97.76
LumB_Specificity	95.45	99.36	98.37	98.33	99.49	100	100	99.23	100	77.88
Normal-like_Specificity	100	100	99.94	100	100	100	100	100	100	100
Basal-like_Pos Pred Value	77.78	89.09	79.94	58.76	72.52	85	94.92	80	100	28.57
Her2E_Pos Pred Value	NA	68	55.97	70.59	21.74	25	71.43	44.12	88.89	14.06
LumA_Pos Pred Value	100	86.48	93.63	63.64	63.73	NA	66.67	71.43	33.33	99.27
LumB_Pos Pred Value	NA	96.36	89.23	90.16	95.24	NA	NA	98.33	100	11.28
Normal-like_Pos Pred Value	NA	100	60	100	100	NA	NA	NA	NA	NA
Basal-like_Neg Pred Value	100	100	99.97	100	100	75	90	100	100	99.86
Her2E_Neg Pred Value	NA	99.44	99.87	96.5	99.22	100	100	99.53	96.77	100
LumA_Neg Pred Value	90	93.17	88.48	98.85	97.18	NA	100	98.32	100	32.75
LumB_Neg Pred Value	NA	88.57	93.19	79.86	75.19	95.83	95.65	69.73	82.35	98.54
Normal-like_Neg Pred Value	90.91	99.01	98.33	87.32	92.42	83.33	97.1	99.18	NA	92.37
Basal-like_Balanced Accuracy	93.33	99.16	98.78	90.85	90.22	72.22	86.62	98.89	100	94.35
Her2E_Balanced Accuracy	NA	95.05	96.89	70.16	82.41	93.48	98.44	92.73	92.83	93.08
LumA_Balanced Accuracy	96.15	88.51	90.31	86.33	87.58	NA	99.25	87.15	91.89	79.07
LumB_Balanced Accuracy	NA	78.17	81.81	68.26	61.65	50	50	75.27	75	74.42
Normal-like_Balanced Accuracy	50	60	52.47	54.35	54.35	50	50	50	NA	50

AIMS vs. PAM50

**Performance**	**GSE60788**	**GSE81538**	**GSE96058**	**GSE25066**	**GSE41998**
Basal-like_Sensitivity	100	100	86.94	91.53	100
Her2E_Sensitivity	100	84.62	77.87	83.78	78.26
LumA_Sensitivity	100	85.9	78.53	54.38	19.78
LumB_Sensitivity	NA	49.52	49.54	58.97	69.7
Normal-like_Sensitivity	50	72.73	88.89	63.64	40.91
Basal-like_Specificity	100	99.14	99.31	97.49	86.98

Her2E_Specificity	100	94.71	97.32	86.41	89.84
LumA_Specificity	91.67	82.33	78.71	98.56	100
LumB_Specificity	100	99	99.13	91.4	94.31
Normal-like_Specificity	100	93.99	86.97	93.75	84.82
Basal-like_Pos Pred Value	100	95	93.71	95.58	83.33
Her2E_Pos Pred Value	100	75.34	76.77	32.63	40.91
LumA_Pos Pred Value	90.91	75.28	78.76	94.57	100
LumB_Pos Pred Value	NA	94.55	94.29	55.42	62.16
Normal-like_Pos Pred Value	100	41.03	32.52	49.12	18.75
Basal-like_Neg Pred Value	100	100	98.47	95.11	100
Her2E_Neg Pred Value	100	96.99	97.48	98.55	97.87
LumA_Neg Pred Value	100	90.31	78.48	82.45	72.03
LumB_Neg Pred Value	NA	84.86	87.13	92.47	95.87
Normal-like_Neg Pred Value	95.24	98.36	99.11	96.45	94.37
Basal-like_Balanced Accuracy	100	99.57	93.13	94.51	93.49
Her2E_Balanced Accuracy	100	89.66	87.6	85.1	84.05
LumA_Balanced Accuracy	95.83	84.11	78.62	76.47	59.89
LumB_Balanced Accuracy	NA	74.26	74.34	75.18	82
Normal-like_Balanced Accuracy	75	83.36	87.93	78.69	62.87

AIMS vs. PAM50none

**Performance**	**GSE60788**	**GSE81538**	**GSE96058**	**GSE25066**	**GSE41998**
Basal-like_Sensitivity	100	100	100	100	100
Her2E_Sensitivity	NA	80.56	86.36	60	71.43
LumA_Sensitivity	84.62	71.62	68.27	56.33	24.29
LumB_Sensitivity	NA	51.61	58.73	49.66	40.48
Normal-like_Sensitivity	0	20	41.67	16.9	34.78
Basal-like_Specificity	86.67	96.91	97.65	80.94	79.89
Her2E_Specificity	95.45	88.08	93.24	83.89	85.66
LumA_Specificity	100	89.62	90.64	99.14	99.52
LumB_Specificity	100	97.76	97.88	96.97	98.46
Normal-like_Specificity	95	90.5	82.38	89.7	84.38
Basal-like_Pos Pred Value	77.78	81.67	77.84	57.46	71.97
Her2E_Pos Pred Value	NA	39.73	37.68	18.95	11.36
LumA_Pos Pred Value	100	89.33	94.19	96.74	94.44
LumB_Pos Pred Value	NA	87.27	85.11	86.75	91.89
Normal-like_Pos Pred Value	0	2.56	4.07	21.05	16.67
Basal-like_Neg Pred Value	100	100	100	100	100
Her2E_Neg Pred Value	NA	97.89	99.31	97.09	99.15
LumA_Neg Pred Value	81.82	72.25	56.25	83.41	79.69
LumB_Neg Pred Value	NA	87.14	91.98	82.82	79.34
Normal-like_Neg Pred Value	90.48	98.91	98.75	86.92	93.51
Basal-like_Balanced Accuracy	93.33	98.46	98.83	90.47	89.95
Her2E_Balanced Accuracy	NA	84.32	89.8	71.95	78.55
LumA_Balanced Accuracy	92.31	80.62	79.46	77.74	61.9
LumB_Balanced Accuracy	NA	74.68	78.3	73.31	69.47
Normal-like_Balanced Accuracy	47.5	55.25	62.02	53.3	59.58

PAM50none vs. PAM50

**Performance**	**GSE60788**	**GSE81538**	**GSE96058**	**GSE25066**	**GSE41998**	**GSE51280**	**GSE58479**	**GSE92977**	**Priedigkeit**	**geicam9906**
Basal-like_Sensitivity	77.78	85.96	72.22	55.03	83.64	94.74	93.44	74.07	100	32.86
Her2E_Sensitivity	0	49.23	41.95	35.14	26.09	50	75	34.15	100	6.32
LumA_Sensitivity	100	99.36	99.59	81.25	74.73	NA	NA	85	33.33	100
LumB_Sensitivity	NA	74.29	61.41	96.15	100	NA	NA	98.68	100	11.88
Normal-like_Sensitivity	0	4.55	4	31.82	77.27	100	25	4.55	NA	15.62
Basal-like_Specificity	100	100	100	100	98.22	100	100	100	100	100
Her2E_Specificity	100	98.82	99.74	96.39	99.61	100	96.92	99.02	96.88	98.91
LumA_Specificity	75	73.09	61.82	91.95	98.94	100	97.1	85.54	100	24.95
LumB_Specificity	100	95	95.72	83.72	79.27	95.83	95.65	75.88	84.85	100

Normal-like_Specificity	90	98.96	98.4	87.72	97.67	95.24	98.46	99.55	100	92.71
Basal-like_Pos Pred Value	100	100	100	100	96.84	100	100	100	100	100
Her2E_Pos Pred Value	NA	88.89	94.81	43.33	85.71	100	60	87.5	88.89	61.11
LumA_Pos Pred Value	76.92	69.82	72.39	82.28	97.14	NA	NA	73.91	100	40.74
LumB_Pos Pred Value	NA	83.87	80.65	51.72	39.29	NA	NA	64.66	58.33	100
Normal-like_Pos Pred Value	0	20	15	19.72	73.91	75	50	50	NA	8.06
Basal-like_Neg Pred Value	86.67	97.75	96.82	78.96	90.22	83.33	66.67	96.9	100	94.06
Her2E_Neg Pred Value	95.45	91.06	93.79	94.98	93.75	95.65	98.44	88.26	100	79.52
LumA_Neg Pred Value	100	99.45	99.34	91.43	89	NA	NA	92.21	83.78	100
LumB_Neg Pred Value	NA	91.35	89.52	99.17	100	NA	NA	99.23	100	70.63
Normal-like_Neg Pred Value	90	94.75	93.55	93.14	98.05	100	95.52	91.39	NA	96.41
Basal-like_Balanced Accuracy	88.89	92.98	86.11	77.51	90.93	97.37	96.72	87.04	100	66.43
Her2E_Balanced Accuracy	50	74.03	70.85	65.76	62.85	75	85.96	66.59	98.44	52.61
LumA_Balanced Accuracy	87.5	86.23	80.71	86.6	86.83	NA	NA	85.27	66.67	62.48
LumB_Balanced Accuracy	NA	84.64	78.57	89.94	89.63	NA	NA	87.28	92.42	55.94
Normal-like_Balanced Accuracy	45	51.75	51.2	59.77	87.47	97.62	61.73	52.05	NA	54.17

* Her2E = HER2-enriched subtype; LumA = Luminal A subtype; LumB = Luminal B subtype.

Table S9. Accuracy in the GSE96058.

**MiniABS vs. PAM50**						
**PAM50**	**MiniABS**					
	**Basal-like**	**Her2E**	**LumA**	LumB	Normal-like	
Basal-like	318	19	13	6	4	360 (10.6%)
Her2E	0	234	57	57	0	348 (10.2%)
LumA	0	1	1703	5	0	1709 (50.1%)
LumB	3	3	404	357	0	767 (22.5%)
Normal-like	3	11	208	2	1	225 (6.6%)
	324<br>(9.5%)	268<br>(7.9%)	2385<br>(70.0%)	427<br>(12.5%)	5<br>(0.1%)	3409
AIMS vs. PAM50						
PAM50	AIMS					
	Basal-like	Her2E		LumB	Normal-like	
Basal-like	313	10	2	0	35	360 (10.6%)
Her2E	17	271	25	17	18	348 (10.2%)
LumA	0	3	1342	6	358	1709 (50.1%)
LumB	3	68	312	380	4	767 (22.5%)
Normal-like	1	1	23	0	200	225 (6.6%)
	334<br>(9.8%)	353<br>(10.4%)	1704<br>(50.0%)	403<br>(11.8%)	615<br>(18.0%)	3409
PAM50 vs. PAM50(none)						
PAM50	PAM50(none)					
	Basal-like	Her2E	LumA	LumB	Normal-like	
Basal-like	260	8	26	15	51	360 (10.6%)
Her2E	0	146	111	91	0	348 (10.2%)
LumA	0	0	1702	7	0	1709 (50.1%)
LumB	0	0	296	471	0	767 (22.5%)
Normal-like	0	0	216	0	9	225 (6.6%)
	260<br>(7.6%)	154<br>(4.5%)	2351<br>(69.0%)	584<br>(17.1%)	60<br>(1.8%)	3409
AIMS vs. PAM50(none)						
PAM50(none)	AIMS					
	Basal-like	Her2E	LumA	LumB	Normal-like	
Basal-like	260	0	0	0	0	260 (7.6%)
Her2E	21	133	0	0	0	154 (4.5%)
LumA	3	95	1605	60	588	2351 (69.0%)
LumB	16	124	99	343	2	584 (17.1%)
Normal-like	34	1	0	0	25	60 (1.8%)
	334<br>(9.8%)	353<br>(10.4%)	1704<br>(50.0%)	403<br>(11.8%)	615<br>(18.0%)	3409
MiniABS vs. PAM50(none)						
PAM50(none)	MiniABS					
	Basal-like	Her2E	LumA	LumB	Normal-like	
Basal-like	259	1	0	0	0	260 (7.6%)
Her2E	4	150	0	0	0	154 (4.5%)
LumA	4	66	2233	46	2	2351 (69.0%)
LumB	14	42	147	381	0	584 (17.1%)
Normal-like	43	9	5	0	3	60 (1.8%)
	324<br>(9.5%)	268<br>(7.9%)	2385<br>(70.0%)	427<br>(12.5%)	5<br>(0.1%)	3409

PAM50(none) is a subtype of the sample identified by the genefu package using the "none" option. * Her2E = HER2-enriched subtype; LumA = Luminal A subtype; LumB = Luminal B subtype.

References

1. Cancer Genome Atlas, N. Comprehensive molecular portraits of human breast tumours. *Nature* **2012**, *490*, 61–70, doi:10.1038/nature11412.
2. Saal, L.H.; Vallon-Christersson, J.; Hakkinen, J.; Hegardt, C.; Grabau, D.; Winter, C.; Brueffer, C.; Tang, M.H.; Reutersward, C.; Schulz, R., et al. The Sweden Cancerome Analysis Network - Breast (SCAN-B)

Initiative: a large-scale multicenter infrastructure towards implementation of breast cancer genomic analyses in the clinical routine. *Genome Med.* **2015**, *7*, 20, doi:10.1186/s13073-015-0131-9.
3. Brueffer, C. Clinical Value of RNA Sequencing–Based Classifiers for Prediction of the Five Conventional Breast Cancer Biomarkers: A Report From the Population-Based Multicenter Sweden Cancerome Analysis Network—Breast Initiative. *J.C.O. Precision Oncology* **2018**, doi:10.1200/PO.17.00135.
4. Hatzis, C.; Pusztai, L.; Valero, V.; Booser, D.J.; Esserman, L.; Lluch, A.; Vidaurre, T.; Holmes, F.; Souchon, E.; Wang, H., et al. A genomic predictor of response and survival following taxane-anthracycline chemotherapy for invasive breast cancer. *JAMA* **2011**, *305*, 1873–1881, doi:10.1001/jama.2011.593.
5. Horak, C.E.; Pusztai, L.; Xing, G.; Trifan, O.C.; Saura, C.; Tseng, L.M.; Chan, S.; Welcher, R.; Liu, D. Biomarker analysis of neoadjuvant doxorubicin/cyclophosphamide followed by ixabepilone or Paclitaxel in early-stage breast cancer. *Clin. Cancer Res.* **2013**, *19*, 1587–1595, doi:10.1158/1078-0432.CCR-12-1359.
6. Anders, C.; Deal, A.M.; Abramson, V.; Liu, M.C.; Storniolo, A.M.; Carpenter, J.T.; Puhalla, S.; Nanda, R.; Melhem-Bertrandt, A.; Lin, N.U., et al. TBCRC 018: phase II study of iniparib in combination with irinotecan to treat progressive triple negative breast cancer brain metastases. *Breast Cancer Res. Treat* **2014**, *146*, 557–566, doi:10.1007/s10549-014-3039-y.
7. Prat, A.; Lluch, A.; Albanell, J.; Barry, W.T.; Fan, C.; Chacon, J.I.; Parker, J.S.; Calvo, L.; Plazaola, A.; Arcusa, A., et al. Predicting response and survival in chemotherapy-treated triple-negative breast cancer. *Br. J. Cancer* **2014**, *111*, 1532–1541, doi:10.1038/bjc.2014.444.
8. Cejalvo, J.M.; Martinez de Duenas, E.; Galvan, P.; Garcia-Recio, S.; Burgues Gasion, O.; Pare, L.; Antolin, S.; Martinello, R.; Blancas, I.; Adamo, B., et al. Intrinsic Subtypes and Gene Expression Profiles in Primary and Metastatic Breast Cancer. *Cancer Res.* **2017**, *77*, 2213–2221, doi:10.1158/0008-5472.CAN-16-2717.
9. Priedigkeit, N.; Hartmaier, R.J.; Chen, Y.; Vareslija, D.; Basudan, A.; Watters, R.J.; Thomas, R.; Leone, J.P.; Lucas, P.C.; Bhargava, R., et al. Intrinsic Subtype Switching and Acquired ERBB2/HER2 Amplifications and Mutations in Breast Cancer Brain Metastases. *JAMA Oncol* **2017**, *3*, 666–671, doi:10.1001/jamaoncol.2016.5630.
10. Bastien, R.R.; Rodriguez-Lescure, A.; Ebbert, M.T.; Prat, A.; Munarriz, B.; Rowe, L.; Miller, P.; Ruiz-Borrego, M.; Anderson, D.; Lyons, B., et al. PAM50 breast cancer subtyping by RT-qPCR and concordance with standard clinical molecular markers. *BMC Med Genomics* **2012**, *5*, 44, doi:10.1186/1755-8794-5-44.

© 2020 by the authors. Submitted for possible open access publication under the terms and conditions of the Creative Commons Attribution (CC BY) license (http://creativecommons.org/licenses/by/4.0/).
